# *Lecanicilliumcauligalbarum* sp. nov. (Cordycipitaceae, Hypocreales), a novel fungus isolated from a stemborer in the Yao Ren National Forest Mountain Park, Guizhou

**DOI:** 10.3897/mycokeys.43.30203

**Published:** 2018-12-04

**Authors:** Ye-Ming Zhou, Jun-Rui Zhi, Mao Ye, Zhi-Yuan Zhang, Wen-Bo Yue, Xiao Zou

**Affiliations:** 1 The Provincial Key Laboratory for Agricultural Pest Management of the Mountainous Region, Institute of Entomology, Guizhou University Guiyang China; 2 Guizhou University, Guiyang 550025, Guizhou, China Guizhou University Guiyang China; 3 Institute of Fungus Resources, Guizhou University, Guiyang, Guizhou 550025, China Guizhou University Guiyang China

**Keywords:** Entomopathogenic fungi, *
Lecanicillium
*, multiple genes, phylogeny, new species

## Abstract

A new species of entomopathogenic fungi, *Lecanicilliumcauligalbarum*, was discovered from a survey of invertebrate-associated fungi in the Yao Ren National Forest Mountain Park in China. The synnemata of this species emerged from the corpse of a stemborer (Lepidoptera), which was hidden amongst pieces of wood on the forest floor. It differs from morphologically similar *Lecanicillium* species mainly in its short conidiogenous cells and ellipsoid to ovoid and aseptate conidia. Phylogenetic analysis of a combined data set comprising ITS, *SSU*, *LSU*, *TEF*, *RPB1* and *RPB2* sequence data supported the inclusion of *L.cauligalbarum* in the *Lecanicillium* genus and its recognition as a distinct species.

## Introduction

The entomopathogenic fungal genus *Lecanicillium* W. Gams & Zare belongs to Ophiocordycipitaceae. It is typified by *Lecanicilliumlecanii* with *Torrubiellaconfragosa* as the sexual morph (Zare and Gams 2001, Wijayawardene et al. 2017). *Lecanicilliumlecanii*was first named as *Cephalosporiumlecanii* Zimm. by Zimmermann in 1898. Viegas incorporated the species in *Verticillium* Nees in 1939 (Gams and Zare 2001). The genus *Verticillium* has a wide host range, including arthropods, nematodes, plants and fungi (Goettel et al. 2008). Zare and Gams (2001) recircumscribed the genus following analyses of morphological data and sequence data for the internal transcribed spacer (ITS) rDNA region (which comprises the ITS1 spacer, 5.8S coding region and ITS2 spacer). All insect pathogens formerly included in *Verticillium* were reclassified in a newly established genus, *Lecanicillium*. In more recent studies, a multilocus nuclear DNA dataset combining sequence data for the nuclear small subunit rDNA (*SSU*), nuclear large subunit rDNA (*LSU*), translation elongation factor 1α (*TEF*), DNA-dependent RNA polymerase II largest subunit (*RPB1*) and DNA-dependent RNA polymerase II second largest subunit (*RPB2*) genes suggests that the genus *Lecanicillium* is paraphyletic (Sung et al. 2007). Phylogenetic analysis of ITS sequence data also supports this conclusion (Sukarno et al. 2009). Kepler et al. (2017) revisited the taxonomic affinities of the Cordycipitaceae (Hypocreales) and proposed that *Lecanicillium* should be rejected because *L.lecanii* is included within the *Akanthomyces* clade and the name *Akanthomyces* Lebert has nomenclatural priority over *Lecanicillium* (Kepler et al. 2017). However, Kepler et al. (2017) transferred to *Akanthomyces* only several species for which sufficient information was available. The phylogenetic affinities of the majority of species in the original circumscription of *Lecanicillium* remain uncertain. Given that there remain unresolved phylogenetic and taxonomic matters concerning *Lecanicillium*, Huang et al. (2018) and Crous et al. (2018) chose to describe new taxa in *Lecanicillium* to avoid creating further confusion in the taxonomy (Crous et al. 2018; Huang et al. 2018).

Presently, 29 *Lecanicillium* species have been formally described and are listed in the Index Fungorum (http://www.indexfungorum.org). Zare and Gams (2001) recognised 14 *Lecanicillium* species based primarily on morphology and ITS sequence data (Zare and Gams 2001). Subsequently, an additional five new *Lecanicillium* species, based on ITS sequence data, were described (Kope and Leal 2006, Sukarno et al. 2009, Kaifuchi et al. 2013). In order to add more sequence information with ITS, Zare and Gams (2008) reassessed the genus *Verticillium* and transferred four species to *Lecanicillium* based on ITS and *SSU* sequence data (Zare and Gams 2008). Except for the *SSU* and ITS gene, more and more researchers have labelled the *Lecanicillium* genus by *TEF* gene. Based on this, two new *Lecanicillium* species were confirmed based on combined with ITS and *TEF* sequence data (Crous et al. 2018). With combined multigene identification of species gradually becoming the convention, two new *Lecanicillium* species were identified based on multilocus (*TEF*, *RPB1*, *RPB2*, *LSU* and *SSU*) sequence data (Park et al. 2016, Chen et al. 2017). *Lecanicilliumsabanense* was identified based on phylogenetic analysis of combined multilocus and ITS sequences (Chiriví-Salomón et al. 2015). *Lecanicilliumsubprimulinum* was identified based on combined analysis of *LSU*, *SSU*, *TEF* and ITS sequence data (Huang et al. 2018).

We carried out a survey of invertebrate-associated fungi in the Yao Ren National Forest Mountain Park near Sandu county in Guizhou province, China. A parasitic fungus was found on a stemborer (Lepidoptera) hiding amongst pieces of wood. Attempting to identify the fungus, we determined it to be a member of *Lecanicillium* but its morphological traits and gene sequences did not correspond with those of any known *Lecanicillium* species. On the basis of its morphology and molecular phylogenetic analysis of multilocus nuclear genes (*TEF*, *RPB1*, *RPB2*, *LSU* and *SSU*) and ITS sequence data, this fungus was suggested to be an unnamed species of *Lecanicillium* and is here described and named *Lecanicilliumcauligalbarum* sp. nov.

## Materials and methods

### Specimen collection and fungus isolation

The specimen was collected from Yao Ren National Forest Mountain Park, Sandu county, Guizhou, China (107°53', 107°58'E; 24°54', 25°59'N, approximately 560–1365 m above sea level), in September 2015 by Yeming Zhou and Xiao Zou. The synnemata of this species emerged from a dead stemborer (Lepidoptera) hidden amongst pieces of wood on the forest floor. The specimen GZUIFR–2015ZHJ and two isolated strains of the fungal asexual stage, GZUIFRZHJ01 and GZUIFRZHJ02, were deposited at the Institute of Fungal Resources of Guizhou University (GZUIFR). The fungal strains were isolated on potato dextrose agar (PDA) medium; one strain was isolated from part of the body and the second strain was isolated from the synnemata.

### Strain culture and identification

The isolated strains were inoculated on PDA at 25 °C for 14 d under 12-h light/12-h dark conditions. The fresh hyphae were observed with an optical microscope (OM, BK5000, OPTEC, USA) following pretreatment with lactophenol cotton blue solution or normal saline.

### DNA extraction, PCR amplification and sequencing

Genomic DNA was extracted using a previously described method (Chiriví-Salomón et al. 2015, Zou et al. 2016). The primers used for PCR amplification of the ITS region, *SSU*, *LSU*, *TEF*, *RPB1* and *RPB2* are listed in Table 1. The PCR reaction conditions employed for each genetic region followed those used in the references listed in Table 1.

To conduct phylogenetic analysis of the sequences obtained, sequences for selected taxa based on recent phylogenetic studies of *Lecanicillium* (Chen et al. 2017, Huang et al. 2018) and Cordycipitaceae (Sung et al. 2007, Kepler et al. 2017, Mongkolsamrit et al. 2018) were downloaded from the National Center for Biotechnology Information GenBank database (https://www.ncbi.nlm.nih.gov/genbank/). A total of 79 accessions of Cordycipitaceae were selected for this study. The sequences used in the study are listed in Table 2.

**Table 1.** Primer information and provenance in this study.

Gene	Primer	Provenance
ITS	F: 5’-TCCGTAGGTGAACCTGCGG-3’	White et al. 1990
	R: 5’-TCCTCCGCTTATTGATATGC-3’	
*SSU*	F: GTAGTCATATGCTTGTCTC	White et al. 1990
	R: CTTCCGTCAATTCCTTTAAG	
*LSU*	F: GTTTCCGTAGGTGAACCTGC	Curran et al. 1994
	R: ATATGCTTAAGTTCAGCGGGT	
*TEF*	F: 5’-GCCCCCGGCCATCGTGACTTCAT-3’	van den Brink et al. 2011
	R: 5’-ATGACACCGACAGCGACGGTCTG-3’	
*RPB1*	F: 5’-CCWGGYTTYATCAAGAARGT-3’	Castlebury et al. 2004
	R: 5’-CAYCCWGGYTTYATCAAGAA-3’	
*RPB2*	F: 5’-GACGACCGTG ATCACTTTGG-3’	van den Brink et al. 2011
	R: 5’-CCCATGGCCTGTTTGCCCAT-3’	

**Table 2.** Specimen information and GenBank accession numbers used in this study.

Species	Voucher Information	ITS	*SSU*	*LSU*	*TEF*	*RPB1*	*RPB2*
* Akanthomyces waltergamsii *	TBRC 7250	MF140749		MF140715	MF140835		
* A. waltergamsii *	TBRC 7251	MF140747		MF140713	MF140833	MF140781	MF140805
* A. sulphureus *	TBRC 7248	MF140758		MF140722	MF140843	MF140787	MF140812
	TBRC 7249	MF140757		MF140721	MF140842	MF140786	MF140734
* A. thailandicus *	TBRC 7246	MF140755		MF140719	MF140840		MF140810
	TBRC 7245	MF140754			MF140839		MF140809
* A. kanyawimiae *	TBRC 7242	MF140751		MF140718	MF140838	MF140784	MF140808
	TBRC 7244	MF140752		MF140716	MF140836		
* A. aculeatus *	HUA 186145		MF416572	MF416520	MF416465		
* A. pistillariaeformis *	HUA 186131		MF416573	MF416521	MF416466		
* A. coccidioperitheciatus *	NHJ 6709	JN049865	EU369110	EU369042	EU369025	EU369067	EU369086
* A. aculeatus *	TS 772	KC519371	KC519368	KC519370	KC519366		
* A. tuberculatus *	BCC16819		MF416600	MF416546	MF416490	MF416647	MF416490
* Ascopolyporus villosus *	ARSEF 6355			AY886544	DQ118750	DQ127241	
* Asc. polychrous *	P.C. 546			DQ118737	DQ118745	DQ127236	
* Beauveria bassiana *	ARSEF 1564	HQ880761			HQ880974	HQ880833	HQ880905
* Bea. brongniartii *	BCC 16585	JN049867	JF415951	JF415967	JF416009	JN049885	JF415991
* Blackwellomyces cardinalis *	OSC 93610	JN049843	AY184974	AY184963	EF469059	EF469088	EF469106
* Bla. cardinalis *	OSC 93609		AY184973	AY184962	DQ522325	DQ522370	DQ522422
* Bla. pseudomilitaris *	NBRC 101409	JN943305	JN941748	JN941393		JN992482	
	NBRC 101410	JN943307	JN941747	JN941394		JN992481	
* Gibellula longispora *	NHJ 12014		EU369098		EU369017	EU369055	EU369075
*Gibellula* sp.	NHJ 7859		EU369107			EU369064	EU369085
	NHJ 10788		EU369101	EU369036	EU369019	EU369058	EU369078
	NHJ 5401		EU369102			EU369059	EU369079
* G. ratticaudata *	ARSEF 1915	JN049837	DQ522562	DQ518777	DQ522360	DQ522408	DQ522467
* Hevansia nelumboides *	BCC 41864	JN201871	JN201863	JN201873	JN201867		
* Hev. novoguineensis *	NHJ 11923		EU369095	EU369032	EU369013	EU369052	EU369072
* Hev. arachnophila *	NHJ 10469		EU369090	EU369031	EU369008	EU369047	
* Hev. cinerea *	NHJ 3510		EU369091		EU369009	EU369048	EU369070
* Lecanicillium acerosum *	CBS418.81	EF641893	KM283762	KM283786	KM283810	KM283832	KM283852
* L. antillanum *	CBS350.85	AJ292392	AF339585	AF339536	DQ522350	DQ522396	DQ522450
* L. aphanocladii *	CBS797.84		KM283763	KM283787	KM283811	KM283833	KM283853
* L. aranearum *	CBS726.73a	AJ292464	AF339586	AF339537	EF468781	EF468887	EF468934
* L. araneicola *	BTCC-F35	AB378506					
* L. araneogenum *	GZU1031Lea		KX845705	KX845703	KX845697	KX845699	KX845701
* L. attenuatum *	CBS402.78	AJ292434	AF339614	AF339565	EF468782	EF468888	EF468935
	KACC42493		KM283756	KM283780	KM283804	KM283826	KM283846
*** L. cauligalbarum ***	**GZUIFRZHJ01**	**MH730663**	**MH730665**	**MH730667**	**MH801920**	**MH801922**	**MH801924**
	**GZUIFRZHJ02**	**MH730664**	**MH730666**	**MH730668**	**MH801921**	**MH801923**	**MH801925**
* L. dimorphum *	CBS345.37		KM283764	KM283788	KM283812	KM283834	KM283854
* L. flavidum *	CBS300.70D	EF641877	KM283765	KM283789	KM283813		KM283855
*L.fungicola var. aleophilum*	CBS357.80	NR_111064	KM283767	KM283791	KM283815	KM283835	KM283856
*L.fungicola var. fungicola*	CBS992.69	NR_119653	KM283768	KM283792	KM283816		KM283857
* L. fusisporum *	CBS164.70	AJ292428	KM283769	KM283793	KM283817	KM283836	KM283858
* L. kalimantanense *	BTCC-F23	AB360356					
* L. lecanii *	CBS101247	JN049836	KM283770	KM283794	DQ522359	KM283837	KM283859
	CBS102067		KM283771	KM283795	KM283818	KM283838	KM283860
* L. longisporum *	CBS102072		KM283772	KM283796	KM283819	KM283839	KM283861
	CBS126.27		KM283773	KM283797	KM283820	KM283840	KM283862
* L. muscarium *	CBS143.62		KM283774	KM283798	KM283821	KM283841	KM283863
* L. nodulosum *	IMI 338014R	EF513012	EF513075				
* L. pissodis *	CBS118231		KM283775	KM283799	KM283822	KM283842	KM283864
* L. primulinum *	JCM 18525	AB712266		AB712263			
	JCM 18526	AB712267		AB712264			
* L. psalliotae *	CBS532.81	JN049846	AF339609	AF339560	EF469067	EF469096	EF469112
	CBS101270		EF469128	EF469081	EF469066	EF469095	EF469113
	CBS363.86		AF339608	AF339559	EF468784	EF468890	
* L. restrictum *	CCF5252	LT548279			LT626943		
* L. sabanense *	JCHA5	KC633232	KC633251	KC875225	KC633266		KC633249
* L. saksenae *	IMI 179841	AJ292432					
* L. subprimulinum *	HKAS99548	MG585314	MG585316	MG585315	MG585317		
	HKAS99549	MG585318	MG585320	MG585319	MG585321		
* L. testudineum *	UBOCC-A112180	LT992874			LT992868		
	UBOCC-A116026	LT992871			LT992867		
* L. tenuipes *	CBS309.85	JN036556	KM283778	KM283802	DQ522341	KM283844	KM283866
* L. uredinophilum *	KACC44082		KM283758	KM283782	KM283806	KM283828	KM283848
	KACC47756		KM283759	KM283783	KM283807	KM283829	KM283849
* L. wallacei *	CBS101237	EF641891	AY184978	AY184967	EF469073	EF469102	EF469119
* Samsoniella inthanonensis *	TBRC 7915	MF140761		MF140725	MF140849	MF140790	MF140815
* Sam. inthanonensis *	TBRC 7916	MF140760		MF140724	MF140848	MF140789	MF140814
* Sam. aurantia *	TBRC 7271	MF140764		MF140728	MF140846	MF140791	MF140818
	TBRC 7272	MF140763		MF140727	MF140845		MF140817
* Sam. alboaurantium *	CBS 240.32	AY624178	JF415958	JF415979	JF416019	JN049895	JF415999
	CBS 262.58	MH857775		MH869308	JQ425685	MF416654	MF416448
* Simplicillium lamellicola *	CBS 116.25	AJ292393	AF339601	AF339552	DQ522356	DQ522404	DQ522462
* Sim. lanosoniveum *	CBS 704.86	AJ292396	AF339602	AF339553	DQ522358	DQ522406	DQ522464
	CBS 101267	AJ292395	AF339603	AF339554	DQ522357	DQ522405	DQ522463
* Sim. obclavatum *	CBS 311.74		AF339567	AF339517	EF468798		

### Sequence alignment and phylogenetic analyses

The DNA sequences used in this study were edited using the LASERGENE software (version 6.0; DNASTAR, Madison, WI, USA). Multiple sequence alignments for *TEF*, *RPB1* and *RPB2* were performed in MAFFT (Katoh and Standley 2013) with the default settings. Multiple sequence alignments for ITS, *LSU* and *SSU* were conducted using MUSCLE algorithm (Edgar 2004) from MEGA 6 (Tamura et al. 2013). The sequences were edited manually. A multiple alignment of the combined partial ITS+*SSU*+*LSU*+*TEF*+*RPB1*+*RPB2* sequences were assembled with MEGA 6 (Tamura et al. 2013) and SEQUENCEMATRIX 1.7.8 (Vaidya et al. 2011). The command ‘hompart’ in PAUP* 4.0b10 was used for assessment of concordance amongst the genes and the ITS region (Swofford 2001). Bayesian inference (BI) was performed using MRBAYES 3.2 (Ronquist et al. 2012) and maximum likelihood (ML) analysis was performed using RAxML (Alexandros 2014) to analyse the combined data which were divided into twelve separate partitions (Kepler et al. 2017; Mongkolsamrit et al. 2018). Two maximum likelihood (ML) analysis and Bayesian inference (BI) analysis were performed. The first analysis was performed as reported by Huang et al. (2018), using the *Simplicilliumlanosoniveum* as the outgroup. The second analysis was performed with *Akanthomyces*, *Samsoniella*, *Blackwellomyces*, *Hevansia*, *Simplicillium*, all the *lecanicillium* and use of *Beauveria* as outgroup (Mongkolsamrit et al. 2018). Nucleotide substitution models were determined by MrModeltest 2.3 (Nylander 2004). For BI, 10 000 000 generations were performed with one tree selected every 500th generation and the GTR+I+G evolutionary model was used. For ML, the model GTRGAMMA was used and a bootstrap analysis with 500 replicates was performed to assess statistical support for the tree topology. Phylogenetic trees were viewed with TREEGRAPH.

## Results

### Sequencing and phylogenetic analysis

**Figure 1.**
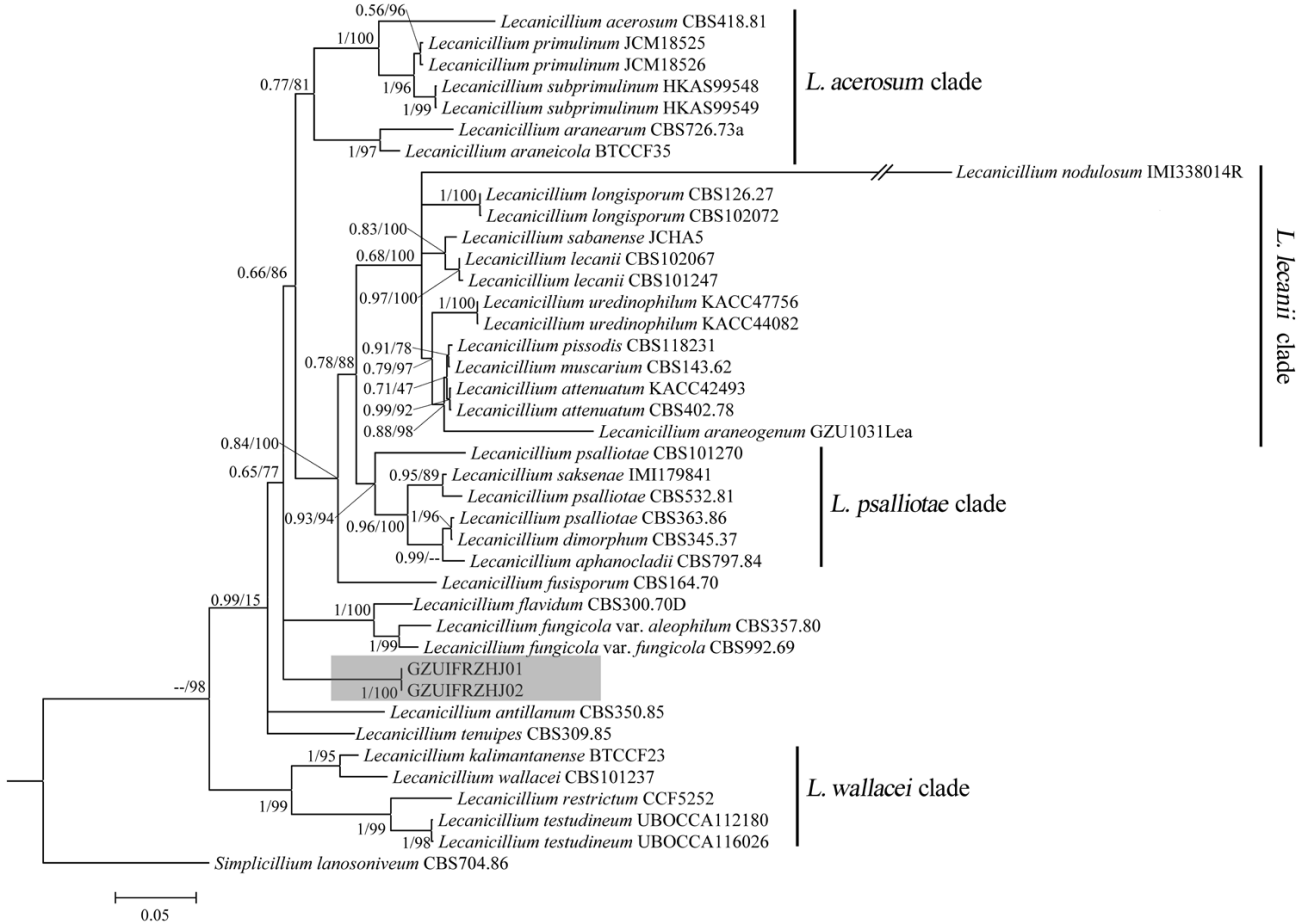
Phylogenetic analysis of the isolated strains GZUIFRZHJ01 and GZUIFRZHJ02 and related species derived from a combined dataset of partial ITS+*SSU*+*LSU*+*TEF*+*RPB1*+*RPB2* sequences. Statistical support values (≥ 0.5/50%) are shown at the nodes for BI posterior probabilities/ML boostrap support.

**Figure 2.**
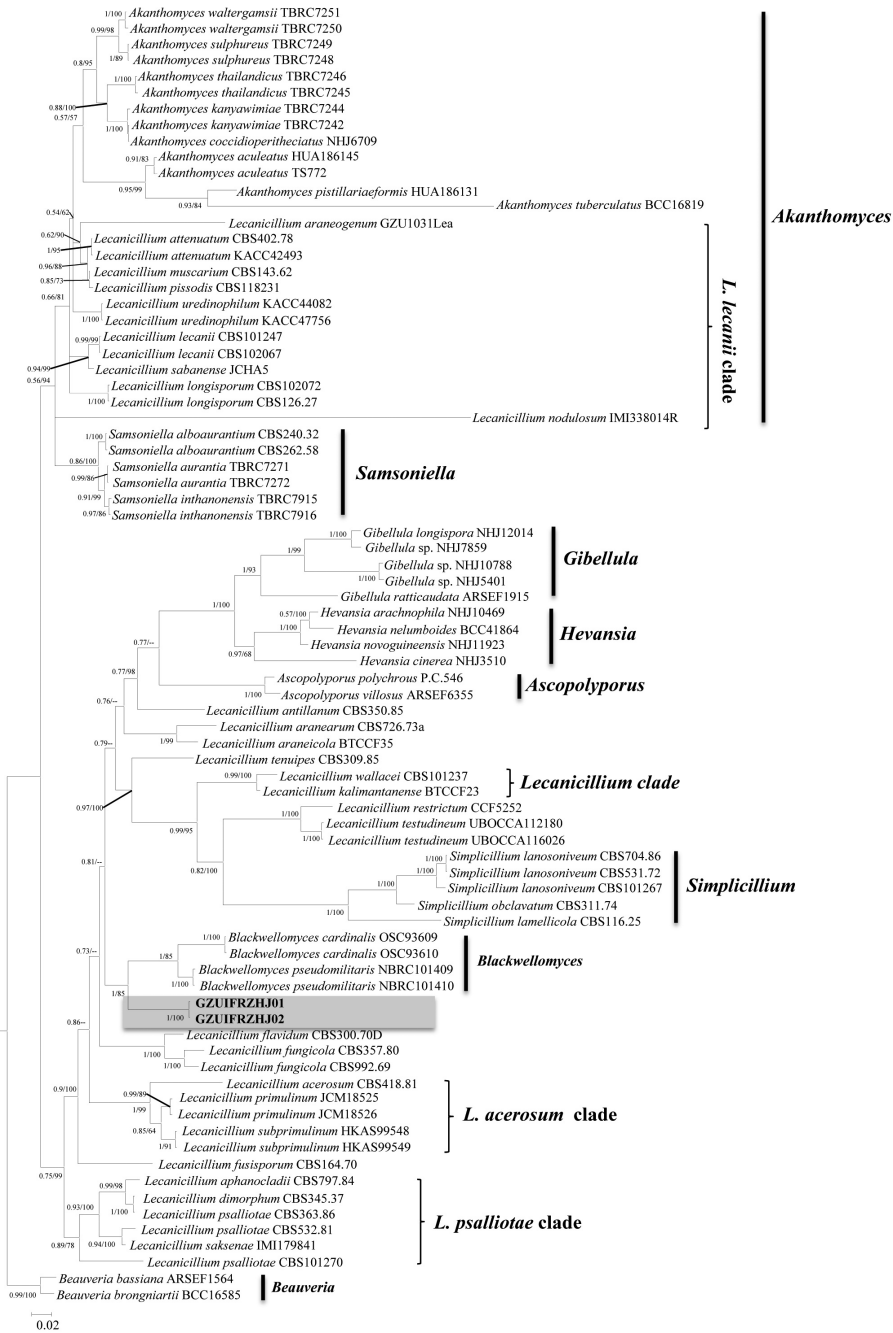
Phylogenetic relationships of the form genus *Lecanicillium*, *Akanthomyces*, *Samsoniella*, *Blackwellomyces*, *Hevansia* and related genera in the Cordycipitaceae. Statistical support values (≥ 0.5/50%) are shown at the nodes for BI posterior probabilities/ML boostrap support.

The first sequence dataset consisted of 3793 bases, including inserted gaps (ITS: 506 bp; *SSU*: 579 bp; *LSU*: 490 bp; *TEF*: 772 bp; *RPB1*: 561 bp; *RPB2*: 885 bp). The second sequence dataset consisted of 2944 bases, including inserted gaps (ITS: 526 bp; *SSU*: 456 bp; *LSU*: 409 bp; *TEF*: 386 bp; *RPB1*: 500 bp; *RPB2*: 667 bp). No significant differences in topology were observed between the BI and ML phylogenies. The first tree formed with almost all the *Lecanicillium* species (only *Lecanicilliumevansii* could not be found in the NCBI) and one *Simplicillium* species (*Simplicilliumlanosoniveum*). The phylogeny was resolved into 4 clades obviously. *Lecanicilliumcauligalbarum* formed an independent branch in a polytomy together with a clade containing *L.flavidum* and *L.fungicola* and a major clade consisting of 27 accessions. The *L.cauligalbarum* lineage received maximum statistical support (BI posterior probabilities 1, ML boostrap 100%), which still remains unnamed (Figure 1). In the second tree, the four *Lecanicillium* clades were also be supported. *Lecanicilliumcauligalbarum* formed an independent branch in a polytomy together with a clade containing *Blackwellomycescardinalis* and *Blackwellomycespseudomilitaris* (BI posterior probabilities 1, ML boostrap 85%) (Figure 2).

## Taxonomy

### Lecanicillium cauligalbarum

Taxon classificationFungiHypocrealesCordycipitaceae

X. Zou, J.R. Zhi & Y.M. Zhou sp. nov.

827984

Figure 3

#### Diagnosis.

Characterised by phialides gradually tapering towards the apex, solitary or 2–3 whorls, 9–14.4 × 1.4–1.8 µm. Conidia cylindric, aseptate, 3.6–6.3 × 0.9–1.8 μm.

#### Type.

CHINA, Guizhou Province, Sandu county (107°53', 107°58'E; 24°54', 25°59'N, approximately 560–1365 m above sea level), September 2015, Yeming Zhou & Xiao Zou. Sequences from isolated strains (GZUIFRZHJ01 and GZUIFRZHJ02) have been deposited in GenBank (accession numbers to be provided).

#### Description.

Colony on PDA 15 mm in diameter after 7 days, 33 mm in diameter after 14 days at 25 °C, colony circular, white, cottony, umbonate, with radiating surface texture from above, with clear radial crack and primrose-yellow from reverse. Mycelium 0.9–1.8 μm wide, hyaline, smooth, septated, branched. Conidiophores usually arising from aerial hyphae, sporulate abundant. Phialides gradually tapering towards the apex, solitary or 2–3 whorls, 9–14.4 × 1.4–1.8 µm. Conidia cylindric, aseptate, 3.6–6.3 × 0.9–1.8 μm. In culture, both phialides and conidia are of similar general shape and size to those found on the host stemborer.

**Figure 3.**
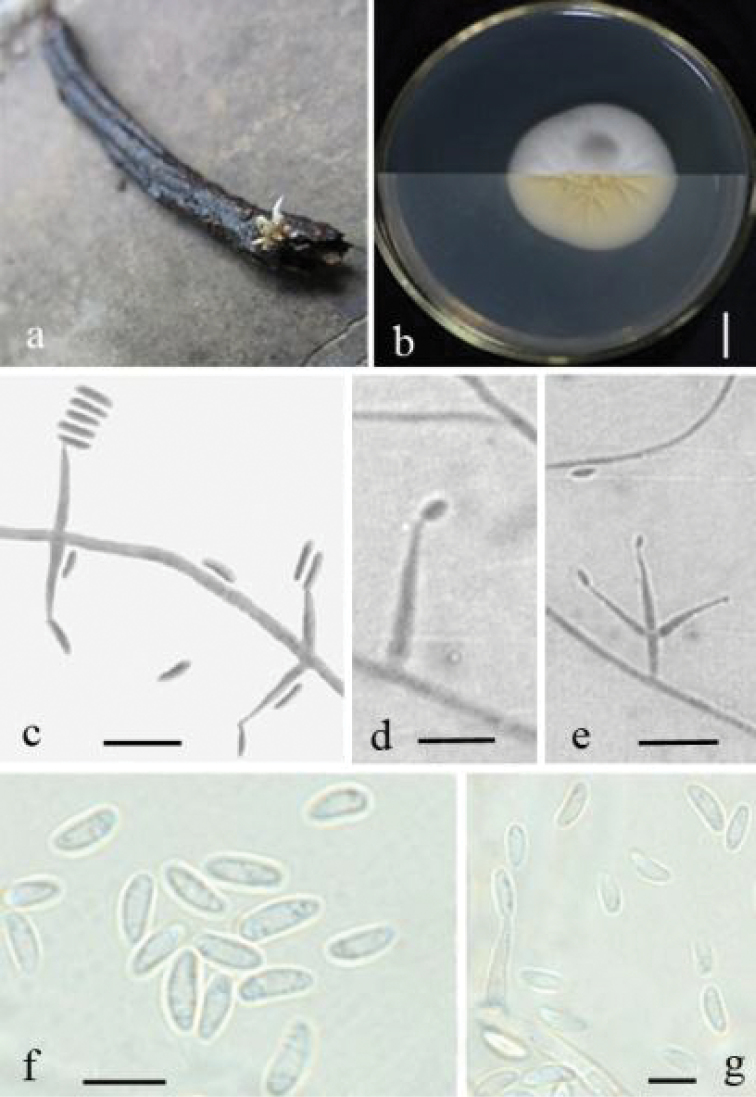
*Lecanicilliumcauligalbarum*. **a** Synnemata emerged from the corpse of a stemborer (Lepidoptera) **b** Culture plate, showing the front (upper) and the back (lower) of the colony, cultured on PDA medium **c–e** Phialides solitary or in 2–3 whorls **f–g** Conidia. Scale bars: 10 mm (**b, c, e**), 5 μm (**d, f, g**).

#### Host.

Stemborer (Lepidoptera) hidden amongst wooden sticks.

#### Habitat and distribution.

Hidden amongst pieces of wood in humid forests of southwest China.

#### Etymology.

The epithet ‘*cauligalbarum*’ refers to the host (stemborer).

#### Teleomorph.

Not known.

#### Remarks.

With regard to phylogenetic relationships, *L.cauligalbarum* is closely related to the *L.fungicola* clade and *L.fusisporum*. The two strains (GZUIFRZHJ01 and GZUIFRZHJ02) formed a distinct lineage. All *Lecanicillium* species were included in the phylogenetic analysis except for *L.evansii* for which sequence data could not be located in public databases, although Zare and Gams (2001) published ITS sequences. The morphological features of *L.evansii* include brownish-cream to brown reverse, phialides solitary or up to 3–4 per node and two types of the conidia, slightly falcate with a pointed end macroconidia 4.5–7.5 × 0.8–1.2 µm and slightly curved microconidia 2.0–3.0 × 0.8–1.2 µm (Zare and Gams 2001). *L.evansii* is distinct from *L.cauligalbarum*, which has conidia of 3.6–6.3 × 0.9–1.8 μm and 9–14.4 × 1.4–1.8 µm phialides.

In morphology *L.cauligalbarum*is is similar to *L.aphanocladii*, *L.attenuatum* and *L.nodulosum* with regard to the short conidiogenous cell (Table 3). However, *L.cauligalbarum* is distinguished by the pattern of spore production and the frequency of the wheel structure.

**Table 3.** Morphological comparison among *Lecanicilliumcauligalbarum* and the other related species.

Species	Colonies	Conidiogenous cell	Conidia	Refrence
* Lecanicillium acerosum *	White, yellow reverse	Solitary or up to 4–5, 30–32×1.8–2.2 μm	Macroconidia fusiform, straight to slightly falcate, 15–20×1.6–2.2 μm, microconidia fusiform, 4.5–7.5×1.0–1.5 μm	Zare and Gams 2001
* L. antillanum *	White, cream-coloured reverse	Solitary or up to 6, subulate, 18–31×1 μm (at the top)	Macroconidia fusiform, 11–18×0.8–1.5 μm, microconidia ellipsoidal, 3–4×0.8–1.2 μm	Zare and Gams 2001
* L. aphanocladii *	White, red, reddish-white to cream-coloured reverse	Solitary, in pairs, verticillate, flask-shaped in the beginning, tapering into a thread-like neck, 4.5–11×1.0–1.8 μm	Solitary, oval to sub-globose, 2.7–4×1.5–2.2 μm	Zare and Gams 2001
* L. aranearum *	White, yellowish-cream reverse	Tapering towards the apex, 20–30×1.2–1.5 μm	Straight or curved, usually asymmetrically narrowed or subacute at the ends, 5–8×0.7–1.5 μm	Zare and Gams 2001
* L. araneicola *	White, creamy-white reverse	Solitary or in whorls of 2–4, slender, tapering toward the tip, (14-)19–31.5×1–2 μm	Macroconidia slightly curved to nearly straight, (7.5-)8.5–12(-14)×1.5–2 μm, microconidia allantoid to ellipsoidal with round ends, 3–5×1–2 μm	Sukarno et al. 2009
* L. araneogenum *	White to light grey, light yellow reverse	Produced in whorls of (1-)2–6(-8), 30–64×1.1–3.2 μm	Forming mostly globose heads, cylindric, 3.2–8.6×1.3–1.6 μm	Chen et al. 2017
* L. attenuatum *	White, yellowish-white reverse	Up to 3–5 per node, 9–15.5×1–2 μm	Cylindrical with attenuate base, 4.5–6.5×1.5–2.0 μm	Zare and Gams 2001
*** L. cauligalbarum ***	White, primrose-yellow reverse	Solitary or 2–3 whorls, 9–14.4×1.4–1.8 μm	Cylindrical, 3.6–6.3×0.9–1.8 μm	**This work**
* L. dimorphum *	White, cream to brownish-cream, red reverse	Two kinds: solitary or 4–5 whorls, 14–30×1.0–1.5μm; short, 5–12×0.7–1.5 μm	Macroconidia falcate with sharply pointed ends, usually evenly curved, 6–11×1.5–2.5μm, microconidia oval to ellipsoidal, 2.5–4.5×1.0–1.5 μm	Zare and Gams 2001
* L. evansii *	White, creamy, brownish-cream to brown reverse,	Solitary or up to 3–4 per node, 20–45×1–1.2 μm	Macroconidia slightly falcate, 4.5–7.5×0.8–1.2μm, microconidia ellipsoidal or curved, 2.0–3.0×0.8–1.2 μm	Zare and Gams 2001
* L. flavidum *	Greyish-white to citron-yellow, citron-yellow reverse	In whorls, 12–35×1.5–2.5 μm, 0.5–1 μm at the tips	Mostly fusiform, long-ellipsoidal to almost cylindrical, slightly sickle-shaped, 4–8×1.5–2 μm	Zare and Gams 2008
L. fungicola var. aleophilum	White, reverse uncoloured	Whorls of 3–10, 15–30×1.5–2.5 μm, 0.5–1.5 μm at the tips	Oblong, fusiform, long ellipsoidal to almost cylindrical, irregular size, 4.5–8×1–2.5 μm	Zare and Gams 2008
L. fungicola var. fungicola	Dirty white, reverse uncoloured	Whorls of 3–7, 14–20(-45)×1.5–3μm, 0.5–1 μm at the tip	Fusiform, long-ellipsoidal to almost cylindrical, sickle-shaped, very unequal size, 4–9(-12)×1.5–2.5(-3.5) μm	Zare and Gams 2008
* L. fusisporum *	White, with red reverse and pigment diffusing	Solitary or up to 5, 16–26×1.0–1.5 μm	Fusiform, straight and rather broad, 3–5×1.5–2.0 μm	Zare and Gams 2001
* L. kalimantanense *	White, creamy-white reverse	Solitary or more often in whorls of 2–5, slender, tapering toward the apex, 12.5–36×1–2 μm	Acerose to fusoid with pointed ends, slightly curved, of varying size, (3.5-)4.5–12×1–2 μm	Sukarno et al. 2009
* L. lecanii *	Yellowish-white, deep yellow reverse	Aculeate and strongly tapering, singly or up to 6, 11–20(-30)×1.3–1.8 μm	Typically short-ellipsoidal, 2.5–3.5(-4.2)×1–1.5 μm, homogeneous in size and shape	Zare and Gams 2001
* L. longisporum *	White to sulphur-yellow, cream-coloured to pale yellow reverse	Tapering towards the apex(sub-aculeate), singly or up to 5–6 or on secondary phialides, 20–40×1.2–2.7 μm	Produced in globose heads, ellipsoidal to oblong-oval, 5.0–10.5×1.5–2.5 μm	Zare and Gams 2001
* L. muscarium *	White, cream-coloured or uncoloured reverse	Solitary or up to 6 (less frequent than in *L.lecanii*), (15-)20–35×1–1.5 μm	Produced in globose heads, ellipsoidal to subcylindrical, more irregular in size and shape, (2-)2.5–5.5(-6)×1–1.5(-1.8) μm	Zare and Gams 2001
* L. nodulosum *	White, cream-coloured reverse	Subulate, up to 6, 10–20×1.5 μm	Produced in heads of about 10μm diam., oval, 2.5–4.5×1.2–1.5 μm	Zare and Gams 2001
* L. pissodis *	White, ceram to yellow reverse	Solitary, up to 3, 16-(18–28)-38×1–2 μm	Up to more than 50 formed in globose droplets, cylindrical to oval, very variable in size and shape, 4–9.2×1.6–2.4 μm	Kope and Leal 2006
* L. primulinum *	Pale yellow, yellowish-brown reverse, brownish-yellow pigment	Solitary or in whorls of 2–5, tapering toward the tip, 20–50(-85)×0.8–1.8 μm	Macroconidia ellipsoidal to cylindrical, 5.0–9.5×1.2–2.5 μm, microconidia oval to ellipsoidal, 3.0–4.8×1.0–2.5 μm	Kaifuchi et al. 2013
* L. psalliotae *	White and red, reddish-cream to cream-coloured reverse, red to purple pigment	Aculeate, solitary or more often 3–4(-6) in whorls on each node, 25–35×1.0–1.5 μm	Macroconidia curved, falcated, 5–10×1.2–1.7 μm, microconidia oval or ellipsoidal, 2.7–3.7×1–1.5 μm	Zare and Gams 2001
* L. restrictum *	Yellowish-white, reverse yellowish-white to pale yellow	Solitary or in whorls of 2–5, tapering toward the tip, (12-)17–30(-36)×0.5–1.5 μm, 0.3–0.5 μm wide on the tip	Macroconidia fusiform or slightly falcate, (5-)6–10(-12)×1–1.5 μm, microconidia ovate, ellipsoidal, obovate or fusoid, frequently slightly curved, 2.5–3×1–1.5 μm	Crous et al. 2018
* L. sabanense *	Pale yellow to duller yellow, orange reverse	Solitary or in whorls of 2–4, 13–19×1.0–2.0 μm, gradually tapering to 0.5–1.0 μm	Forming mostly globose heads, 9–20 μm diam, ellipsoidal to ovoid, 3.5–4.5×1.5–2.0 μm	Chiriví-Salomón et al. 2015
* L. saksenae *	White, creamy white reverse	Solitary or often in whorls of 2–4, slender, tapering towards the apex, 14.5–36×1.0–2.0 μm	Macroconidia slightly curved, 6–13×1.5–2 μm, microconidia ellipsoidal to fusoid with round ends, nearly straight to slightly curved, 2.5–5×1.5–2 μm	Sukarno et al. 2009
* L. subprimulinum *	Creamy, primrose-yellow reverse	Tapering towards apex, discrete, solitary or up to 2–3 per node, 19–32×1.5–3.5 μm	Ovoid to ellipsoidal, elongated, straight or slightly curved, 4–15×2–6 μm	Huang et al. 2018
* L. testudineum *	White, centrally raised, wrinkled, reverse pale yellow to greyish-yellow	Solitary or in whorls of 2–4, tapering toward the tip, (13-)16–45(-53)×0.5–1 μm (exceptionally 80 μm long), 0.5–1 μm wide on the tip	Macroconidia fusiform or slightly falcate, 3.5–6(-6.5)×1–1.5 μm, microconidia ovate, ellipsoidal or fusoid, curved to reniform, 2–3.5×1–1.5 μm	Crous et al. 2018
* L. tenuipes *	White, reverse uncoloured	Arising singly or in scanty whorls, 20–35(-40)×1.2–1.5 μm	Microconidia ellipsoidal, straight, 3.0–5.5(-6.5)×1.0–1.5 μm, microconidia fusiform to falcate, 8–17×1.5–1.8 μm	Gams et al. 1984; Zare and Gams 2001
* L. uredinophilum *	White to cream coloured, reverse cream coloured	Produced singly or in whorls of up to 3–5, 20–60×1–2.5(-3) μm	Cylindric, oblong or ellipsoid, 3–9×1.8–3 μm	Park et al. 2016
* L. wallacei *	White, cream-coloured to creamish-brown reverse	Sollitary or up to 3–4, aculeate, (14-)17–25(29)×0.7–1.2 μm	Macroconidia, fusiform to falcate, (7.0-)8.5–10.5(-12.5)×1.0–1.5 μm, microconidia ellipsoidal to slightly falcate, (3.0-)4.0–5.5(-6.5)×0.7–1.2 μm	Zare and Gams 2001, 2008

## Discussion

The genera *Lecanicillium* and *Simplicillium* belong to the Cordycipitaceae (Sung et al. 2007). The two genera are indistinguishable in morphological traits (Sung et al. 2001; Zare and Gams 2001). However, *Lecanicillium* and *Simplicillium* are clearly separated in molecular phylogenetic analyses (Kouvelis et al. 2008; Maharachchikumbura et al. 2015; Nonaka et al. 2013). As an insect pathogen, *Lecanicillium* spp. has potential for development as effective biological control agents against a number of plant diseases, insect pests and plant-parasitic nematodes (Goettel et al. 2008). Fifteen commercial preparations based on *Lecanicillium* spp. have been developed or are in the process of being developed (Faria and Wraight 2007). Kepler et al. (2017) concluded that *Lecanicillium* should be incorporated into *Akanthomyces* and formally transferred a number of *Lecanicillium* species. However, the compatibility of *Lecanicillium* was not so good in this study. Species that have been transferred to *Akanthomyces* were all assembled in the *L.lecanii* clade in the present study. The remaining species included in the present analyses were divided into multiple clades similar to those retrieved by Kepler et al. (2017). Relationships amongst *Lecanicillium* species thus appear to be more complicated than expected. Thus, we also prefer to describe the new taxon as a *Lecanicillium* species, consistent with Huang et al. (2018), owing to the uncertainty in generic boundaries.

In a comparison of all *Lecanicillium* species included in the present study, we were unable to identify morphological synapomorphies that characterise the phylogenetic groups. However, the species that show a close phylogenetic relationship are more similar in morphology than those that are phylogenetically distant. For example, the *L.lecanii* clade, which has globose heads with a higher number of conidia, are distinguishable from those clades that usually have one conidium visible at the top of the phialide in the phylogenetic tree presented here. In our phylogeny study, the node connecting *L.antillanum* and *L.tenuipes* is the basal node for the major clade. So the relationships of all of the lineages involved may change with more data or a different dataset. Therefore, more species are needed to enrich the phylogenetic study of *Lecanicillium* spp.

We know that *Lecanicillium* has a different origin into the Cordycipitaceae. We consider that the ones ‘*L.lecanii* clade’ in pig.1 form a strong clade inside of *Akanthomyces*. Maybe all these should be moved to the *Akanthomyces* including *Lecanicilliumlongisporum*. In addition, the elimination of the genus may create more chaos considering the unsolved other clades.

*Blackwellomyces* Spatafora & Luangsa-ard is diagnosed by the unique characters of the ascospore, which have irregularly spaced septa and do not disarticulate into part-spores at maturity as advised by Kepler et al. (2017). It includes *Blackwellomycescardinalis* and *Blackwellomycespseudomilitaris*. Asexual morphs have been described as similar to species in *Clonostachys*, *Hirsutella*, *Isaria* and *Mariannaea* (Hywel-Jones 1994; Sung and Spatafora 2004). Although the new species are close to the *Blackwellomyces* in the phylogenetic tree, we think they are clearly distinguished from *Blackwellomyces* by the morphology. We also treat the new species as *Lecanicillium* considering the small sample and the unknown teleomorph.Thus, based on the present molecular phylogeny, derived from nuclear and ribosomal DNA sequence data, together with morphological evidence, a distinct new *Lecanicillium* species, *L.cauligalbarum*, is proposed.

## Supplementary Material

XML Treatment for Lecanicillium cauligalbarum
